# The Diagnostic Significance of Certain Signs and Symptoms in Joint Disease

**Published:** 1894-04-28

**Authors:** 


### THE DIAGNOSTIC SIGNIFICANCE OF CERTAIN SIGNS AND SYMPTOMS IN JOINT DISEASE.

*Swelling.*—This is recognised with much greater facility in some joints than in others. In the knee, for instance, we can readily detect its presence; in the hip, on the other hand, buried as it is beneath the muscles, it may be present and yet unrecognised. In the elbow, although the front of the joint is deeply placed beneath muscles, yet swelling can readily be detected behind, around the olecranon. In the ankle the joint is bounded on either side by the malleoli, and behind we cannot get down to the joint because of the tendo-achillis, so that it is in the anterior aspect, under the extensor tendons, we have to look for the swelling in disease of this articulation. In the fingers it is on the dorsal surface, where the tendinous structures covering them are thinnest, that we must expect to find the swelling most marked when the joints are affected; and in the wrist the swelling is generally more perceptible on the dorsal surface, as on this aspect the joint is less thickly covered than on the front.

And now with regard to the character of the swelling. It may be soft and fluctuating, but this does not always mean fluid. The soft, pulpy, tuberculous material in joints has often a fluctuating feeling. We object to the term semi-fluctuating as applied to solid tissues so soft as to lead to the supposition it is fluid from palpation. For instance, a fatty tumour or a soft sarcoma may give to the fingers precisely the sensation fluid would. It is fluctuation, not semi-fluctuation. We may like to call it semi-fluctuation when we find it is a soft, solid growth, and not fluid, but really it is not in many cases possible by palpation alone to distinguish a very soft solid from a fluid swelling, and it certainly is not in many pulpy joints. Moreover, the difficulty of diagnosis is increased by the fact that in some cases of tubercular joints, abscess, or more accurately local caseous breaking down, occurs in the pulpy tissue, which does not communicate with the joint, and hence in fluctuating or almost fluctuating tissue around joints we may have true fluid purulent collections not communicating with the joint cavity. Hence we should verify our diagnosis in doubtful cases by exploratory puncture with a carefully carbolised exploring syringe, for on the one hand we may otherwise cut into simply soft fluctuating pulpy tissue, and on the other we must remember that in this tissue abscess does occur at times, and be on the look-out for it by noting any rapid increase with a greater sense of fluctuating than in the other parts of the swollen tissues, and any commencing redness of the skin over it. The character of the swelling due to tubercular disease in the synovial membrane of the joint is a slow obliteration of the sharper outlines of the bones forming the joint. The grooves and depression get filled up, and the ends of the bones look and feel larger than normal from the swelling of the soft tissues over them. There is hardly any tenderness and very little pain in an early stage.

And now let us contrast with this pulpy tubercular swelling the swelling due to the presence of free fluid in the joint, and let us take the knee as an example. If the fluid is free in the joint, we shall get fluctuation across the joint, not simply at various spots in the swelling. In pulpy tissue we do not, as a rule, get fluctuation with the fingers widely separated: in cases of free fluid in the joint a wave is communicated right across the articulation. Again, the fluid if in large amount will certainly raise the patella from the condyles of the femur when the limb is extended. This is best appreciated by obtaining what is known as the "patella bob." A smart push is given to the patella downwards, and it is felt to suddenly strike the femoral condyles, from which it would not be separated were there no fluid in the joint. If the joint is not much distended with fluid, it may collect in the sides of the joint and in the upper synovial pouch, and so not raise the patella, but its presence can often be demonstrated in these cases by simply pressing it out of this upper synovial pouch by firmly drawing the hand down the thigh towards the patella, and then the fluid will raise that bone and the "patella bob" can be easily obtained. Of course where there is very little free fluid in the joint there may not be enough to do this, and we shall have to be guided by the feeling of very soft partial distension of the upper synovial sac and at the sides of the patella.

In cases of fractures into the knee-joint—fractured patella, for instance—the swelling of the joint which at once occurs is due to hæmorrhage into the joint, and the same may occur in "bleeders" without injury, but almost always the fluid is serum or pus. Serous effusion may be due to injury—blows or falls on the joint, or due to rheumatism, or to miliary tubercles scattered over the synovial membrane, or no apparent cause may be discoverable, except the common condition of exposure to cold. Pus in the joint may be due to disease in the articular ends of the bones spreading to the joint, a septic wound of the joint, or may be pyæmic in origin, or may accompany or follow certain diseases, such as scarlet fever. If the pain is very severe, the skin very red over the joint, the temperature very high, and the constitutional disturbance severe, we may suspect pus rather than serum, and make an absolute diagnosis by means of an aseptic exploring syringe, but it must be thoroughly aseptic or we may readily convert a serous effusion into a purulent one, with perhaps destruction of the joint from arthritis. But we must not forget that in gonorrhœal rheumatism the pain, redness of the skin, and the constitutional disturbance from loss of sleep is sometimes extreme and yet no pus, hardly any fluid of any kind is present in the joint. Nor must we forget that in acute rheumatism the pain, redness of skin, and high temperature may be very marked, but here the lesion is a multiple one. It is rather where a single joint becomes more and more distended with fluid, more and more painful and red, with rising temperature, and some common cause of suppurative arthritis exists, as a puncture with a septic nail or knife, or where symptoms and signs of chronic abscess in the head of a neighbouring bone have existed some time, that we should suspect the fluid present in the joint to be pus. Of course, in advanced tubercular joint disease pus forms in the joint, but here the history of the case will guide us.

As in cases of fluid in the pleura, so in fluid in the joints we have much difficulty in certain cases in coming to a conclusion as to whether it is serous or purulent, and as in the chest so here we must settle the question by the use of a thoroughly carbolised exploring syringe. It may be as well to boil the needle of the syringe with a solution of caustic potash first to remove any dried pus which might have been imperfectly removed in cleaning.

